# SPRD: a surface plasmon resonance database of common factors for better experimental planning

**DOI:** 10.1186/s12860-021-00354-w

**Published:** 2021-03-06

**Authors:** Purushottam B. Tiwari, Camelia Bencheqroun, Mario Lemus, Taryn Shaw, Marilyn Kouassi-Brou, Adil Alaoui, Aykut Üren

**Affiliations:** 1 Department of Oncology, Georgetown University, Washington, DC 20057 USA; 2 Innovation Center for Biomedical Informatics (ICBI), Georgetown University, Washington, DC 20057 USA; 3 Geisel School of Medicine, Dartmouth College, NH 03755 Hanover, USA

**Keywords:** Surface plasmon resonance (SPR), Sensor chip, Analyte-ligand interactions, And equilibrium dissociation constant

## Abstract

**Background:**

Surface plasmon resonance is a label-free biophysical technique that is widely used in investigating biomolecular interactions, including protein-protein, protein-DNA, and protein-small molecule binding. Surface plasmon resonance is a very powerful tool in different stages of small molecule drug development and antibody characterization. Both academic institutions and pharmaceutical industry extensively utilize this method for screening and validation studies involving direct molecular interactions. In most applications of the surface plasmon resonance technology, one of the studied molecules is immobilized on a microchip, while the second molecule is delivered through a microfluidic system over the immobilized molecules. Changes in total mass on the chip surface is recorded in real time as an indicator of the molecular interactions.

**Main body:**

Quality and accuracy of the surface plasmon resonance data depend on experimental variables, including buffer composition, type of sensor chip, coupling chemistry of molecules on the sensor surface, and surface regeneration conditions. These technical details are generally included in materials and methods sections of published manuscripts and are not easily accessible using the common internet browser search engines or PubMed. Herein, we introduce a surface plasmon resonance database, www.sprdatabase.info that contains technical details extracted from 5140 publications with surface plasmon resonance data. We also provide an analysis of experimental conditions preferred by different laboratories. These experimental variables can be searched within the database and help future users of this technology to design better experiments.

**Conclusion:**

Amine coupling and CM5 chips were the most common methods used for immobilizing proteins in surface plasmon resonance experiments. However, number of different chips, capture methods and buffer conditions were used by multiple investigators. We predict that the database will significantly help the scientific community using this technology and hope that users will provide feedback to improve and expand the database indefinitely. Publicly available information in the database can save a great amount of time and resources by assisting initial optimization and troubleshooting of surface plasmon resonance experiments.

## Background

Surface plasmon resonance (SPR) is one of the most commonly used label-free biophysical techniques that can provide real-time information on interaction of two molecules [1–3]. There have been many successful commercial applications of this technology. Several applications using sensorchips made of thin continuous gold layer includes but not limited to Biacore (Cytiva), BI (Biosensing instruments), ProteOn (Bio-Rad), Carterra LSA (Carterra), Reichert 4/2SPR (Xantec Bioanalytics), Sierra SPR-32/24 Pro (Bruker), MP-SPR Navi (BioNavis), Pioneer (ForteBio), P4SPR (Affinite Instruments). Besides, conventional SPR on the continuous metal surface, there are other biosensing applications based on nanoparticles as sensors that include but not limited to OpenSPR (Nicoya) and SoPRano (BMG LABTECH) using localized surface plasmon resonance (LSPR) technology [4] as well as INB-D200 (INSTANT NanoBiosensors) using fiber optic particle plasmon resonance (FOPPR) [5] utilizing SPR effects. The SPR technique is very useful in SM binding [6, 7], hit validation [7], and lead identification [8] in drug discovery via the detection of direct molecular interactions [9]. One of the two interacting partners is immobilized on the metal chip surface (ligand) whereas the other binding partner in solution (analyte) is flown over the ligand-immobilized surface [10]. On the other side of the metal chip, an incident light is directed to the ligand free surface at an angle to achieve total internal reflection and the reflection angle is recorded in real-time. Upon binding of the analyte to the ligand, due to the change in mass, the refractive index changes [11, 12]. This change in the refractive index corresponds to the SPR signal, which can be calculated as a change in total mass on the chip surface and the data is presented as SPR sensorgrams [13]. SPR data can be fitted to physical equations to determine the kinetics rate constants, association rate constant (k_a_ or k_on_), dissociation rate constant (k_d_ or k_off_), and equilibrium dissociation constant (K_D_) of different types of biomolecular interactions, including monophasic and biphasic interactions [14–18].

The quality of the SPR data depends on the several external factors such as buffer compositions [19], types of the sensor chip with different matrix on the gold surface [20–22], and coupling techniques of ligands on the sensor surface [23, 24]. For slow dissociating analyte-ligand interactions, the chip surface has to be regenerated with proper regeneration solution without affecting the ligand activity [25]. Therefore, several factors have to be considered and optimized to generate good quality SPR data. Experiments initially may require a big investment of time and resource to find the optimum conditions for a specific pair of ligand and analyte that are being studied. Many labs will go through a trial and error process to troubleshoot these steps based on their experience with similar molecules. New users of this technology and experienced users dealing with a completely new class of molecules spend valuable time and research funding to determine the best approach.

Here, we introduce a SPR database (SPRD) with searchable experimental details from 5140 publications. These experimental factors can be searched within the database and are very helpful for users to design new experiments, which are related to particular biomolecules already used in the previous experiments. Although we focused to gather information from publications with conventional SPR systems using sensorchips made of continuous thin metal layer, the information included in our database is also useful for researchers using other instruments based on derivative techniques such as LSPR and FOPPR to investigate biomolecular interactions. The searchable experimental factors include, ligand, analyte, sensorchip, running buffer, and regeneration solution. The SPRD will save significant amount of time and resources that would have been invested at the beginning of SPR experiments to figure out the optimal experimental conditions and potential troubleshooting. The database is designed to be corrected, updated, and expanded by input from its users.

## Construction and content

### Literature included in SPRD

Experimental details in the SPRD were assembled as 5500 entries in the database from a library of 5140 manuscripts containing SPR studies. The library was compiled through using key words in different search engines. We used “Biacore” keyword in PubMed (https://pubmed.ncbi.nlm.nih.gov/). We also included manuscripts that were evaluated in five review articles on SPR [26–30]. These searches resulted in ~ 8200 publications. Review articles and papers with theoretical data without experimental validation were excluded. Manuscripts that did not describe SPR experiments with enough detail were also excluded. Only the manuscripts written in English were included. We were limited with availability of full text manuscript based on subscription list of Georgetown University library. Therefore, the final number of manuscripts we could include in the library was 5140 in November 2020.

### Data entry

Each manuscript in the library was read by one of the authors and technical details about the SPR experiment were entered and managed in Research Electronic Data Capture (REDCap) electronic data capture tool hosted at Georgetown University [31, 32]. If available, the following details were recorded for each manuscript; name of the ligand(s), name of the analyte (s), ligand’s protein tag, analyte’s protein tag, ligand class (protein, DNA, RNA, SM etc.), analyte class (protein, DNA, RNA, SM etc.), type of sensorchip, name of SPR instrument, ligand immobilization or capture method, immobilization or capture buffer, immobilization or capture running buffer, ligand immobilization or capture level, kinetics running buffer, kinetics regeneration solution, k_a_, k_d_, and K_D_. If ligands were captured using intermediate molecules immobilized on the sensor surface first, we also captured the name, immobilization method, immobilization buffer, immobilization running buffer, and immobilization level of the intermediate molecule. For each set of data entry, we provided the PubMed unique identifier number (PMID) and a hyperlink to it. If there were more than two entirely different interaction types found in the same publication, we captured each information as an independent entry. Therefore, the SPRD has 5500 entries from the library of 5140 different publications. If the publication reported different mutants of the same ligand or analyte, we only captured information related to the wild-type form.

### Utility and discussion

SPR experiments can be designed to get binary results to evaluate if a ligand directly binds to an analyte or not. They can also be designed to screen analyte libraries composed of SMs, peptides or oligos. A significant number of experiments seek to identify the kinetics parameters (k_a_, k_d_, K_D_) of the specific analyte-ligand interaction. Both the binary results and the kinetics parameters determined by any SPR experiment may significantly vary depending on buffer conditions, chip types, and ligand immobilization procedures. For example, the K_D_ values of interactions between wilt-type Eap45 GLUE domain and ubiquitin were found to be ~ 411 μM and ~ 261 μM in phosphate and tris buffers, respectively [19]. Likewise, the K_D_ value for Factor H (fH) binding to C3b was found to be about three times higher using a CM5 chip (~ 2.2 μM) when compared to using a C1 chip (~ 0.7 μM), using the same ligand coupling chemistry and buffer condition [22]. Unlike CM5, the C1 chip does not have a dextran matrix coated on the sensor surface [33]. It is to be noted that the lower ligand immobilization level used for the C1 chip (140 RU) as compared to the CM5 chip (384 RU) might also be the contributing factors for the change in the K_D_ values in this study [22]. Moreover, when PD-1was immobilized on a CM5 chip by amine coupling [23] or biotin-tagged PD-1 was captured on a streptavidin (SA) coated chip [24], calculated K_D_ value for binding to PD-L1 changed approximately 28 fold (~ 0.9 μM [23] vs. ~ 25 μM [24]). In this example, difference in the sources of interacting partners (PD-1 and PD-L1) may have contributed to the difference in K_D_ values too. There is a variation in reported K_D_ values for antigen-antibody interactions when immobilization method of antibody (ligand) using standard amine coupling chemistry changed to the capture of the antibody (ligand) using a secondary antibody immobilized on the same chip surface [34]. Therefore, the results from the same ligand and analyte interaction can be completely different based on simple experimental conditions. A user who wants to start a new SPR experiment for an analyte-ligand pair faces a number of choices for the chip, capture method, and buffer conditions. Testing all possible combinations of these factors is not practical and not feasible in some cases. Therefore, we decided to generate the SPRD to assist future users of SPR technology to take full advantage of the collective knowledge in SPR literature.

When it was clearly stated in the original publication, we recorded the following details related to SPR experiments in the SPRD:

#### Reference

We present the title of the publication, PMID and the hyperlink to PubMed for each data entry. SPRD users can reach to the original publication to see the entire details of the SPR experiments and relevant information on the source of key materials.

#### Ligand name

The name of the ligand was entered as it appeared in the original paper. If the ligand is a protein, its alternative names can be searched in GeneCards hyperlink (www.genecards.org) that is provided in the SPRD results page. If the ligand name was not provided in the original manuscript, the entry was listed under “Undefined”.

#### Other ligands used

If a publication had multiple ligands that were immobilized in the same experiments using a similar coupling chemistry, we captured their names in the same record. However, the SPRD does not have detailed kinetics information for each ligand. We recommend users to follow the reference for details.

#### Analyte name

This corresponds to the name of the analyte that was flowed over the ligand-immobilized surface. The database entry has subsequent information related to the binding of this single analyte to the ligand for each entry. If the analyte is a protein, its alternative names can be searched in GeneCards hyperlink (www.genecards.org) that is provided in the SPRD. If the analyte name was not provided in the original manuscript, the entry was listed under “Undefined”.

#### Other analytes used

If a publication presented multiple analytes that were also flowed over the same ligand-immobilized surface, we captured their names. The SPRD does not have detailed kinetics information for each analyte. We refer users to the original manuscript for details.

#### Ligand tag

Some ligands had tags for easier purification or immobilization. Commonly used protein purification tags are 6xHis, GST, FLAG and Mcy. When they were used, their names were included under this category.

#### Analyte tag

They were captured similar to ligand tags.

#### Ligand class

Each ligand was assigned to class of molecules, which included protein, DNA, RNA, SM, peptide, and antibody. If the class of the ligand did not fit any of these, it was entered as “other”. If the ligand class was not provided in the original manuscript, the entry was listed under “Undefined”.

#### Analyte class

Analyte classes were captured similar to ligands. If the analyte class was not provided in the original manuscript, the entry was listed under “Undefined”.

#### Sensorchip used

We recorded the type of sensorchip used in the SPR experiments as mentioned in each publication. The types of sensorchips included C1, CM3, CM4, CM5, CM7, HPA, L1, NTA, PEG, and SA chip. Since majority of the experiments were using Biacore instruments, the chip categories were matched to its manufacturer’s nomenclature. If the type of the sensorchip was not any of the types mentioned above, we entered the chip type(s) as “other”. If the chip information was not provided in the original manuscript, the entry was listed under “Undefined”.

#### Instrument used

We recorded the name of the commercial biosensor instrument that was used to run the SPR experiment.

#### Immobilization or capture method

If the immobilization or capture method was mentioned in the publication, we recorded that information. These methods included, immobilization using the amine coupling chemistry (amine coupling), the capture of His-tagged ligand on a nickel chelated NTA surface (Ni^2+^-NTA capture) or anti-His antibody immobilized surface (anti-His capture), the capture of GST-tagged ligands on an anti-GST antibody immobilized surface (anti-GST capture), the capture of biotin-tagged ligands on a streptavidin- or neutravidin-coated surface (biotin-capture), immobilization using thiol coupling chemistry (thiol coupling), immobilization using maleimide chemistry (maleimide coupling), and capture of ligands on ligand specific antibody immobilized surface (antibody capture). If the immobilization or capture method mentioned in the publication was not any of the above-mentioned methods, we recorded that information as “other”. If the capture method was not explained in the original manuscript, the entry was listed under “Undefined”.

#### Immobilization or capture buffer

We recorded the composition and pH information of the buffer in which the ligand was diluted for immobilization step.

#### Immobilization or capture running buffer

We recorded the composition and pH information of the buffer, which runs in the background during immobilization or capture of the ligand. In many experiments, this buffer was different from the immobilization or capture buffer in which the ligand was diluted directly.

#### Immobilization or capture level

We recorded the response amplitude obtained when ligands were immobilized or captured on the sensor surface. The unit of this amplitude varies depending on the SPR-based biosensor instrument. In Biacore instruments, which were the most common tool, it was response unit (RU).

#### Intermediate molecule

In some experiments, ligands were captured on the surface on which another molecule was already immobilized. For example, in anti-His capture of His-tagged ligand, the anti-His antibody was first immobilized on the chip surface and then the His-tagged ligand was flowed through. Here the anti-His antibody was considered as the intermediate molecule. If this molecule was required in a particular experiment and based on the availability of the information in the publication, we recorded class, immobilization method, immobilization buffer and pH, immobilization running buffer and pH, and immobilization level for the intermediate molecule as we have done that for ligands.

#### Kinetics running buffer

This is the buffer that was used to dilute analyte before it was injected into SPR instruments and the same buffer that runs in the background. We recorded the composition and pH of the kinetics running buffer.

#### Kinetics regeneration solution

Very strong analyte-ligand interactions have small k_d_ values, which indicates a very slow dissociation of analyte form the ligand. Since the sensor surface has to be regenerated for the next cycle of the analyte injection, a regeneration solution is used to dissociate the analyte-ligand complex without damaging the activity of the ligand on the chip surface [25]. We recorded the composition and pH of the regeneration solution.

#### k_a,_ k_d,_ and K_D_

We recorded the association rate constant (k_a_ or k_on_), dissociate rate constant (k_d_ or k_off_), and the equilibrium dissociation constant (K_D_) of the interactions between the ligand and analyte.

### SPRD web portal

We designed and implemented a publicly accessible and searchable web portal available to query current resources and information in the collected and curated data repository based on different experimental factors and data elements captured in the database. The portal features additional capabilities that include:

- Interactive dashboards
- Search based on the recorded features (ligand, analyte, chip used, immobilization method used or reference)
- Access publications in PubMed – search based on regular expression, matching characters or PubMed’s Identifier (PMID)
- Submit and report errors (forms)
- Submit new entries – for the team to assess and validate before making them public.

**Fig. 1:**
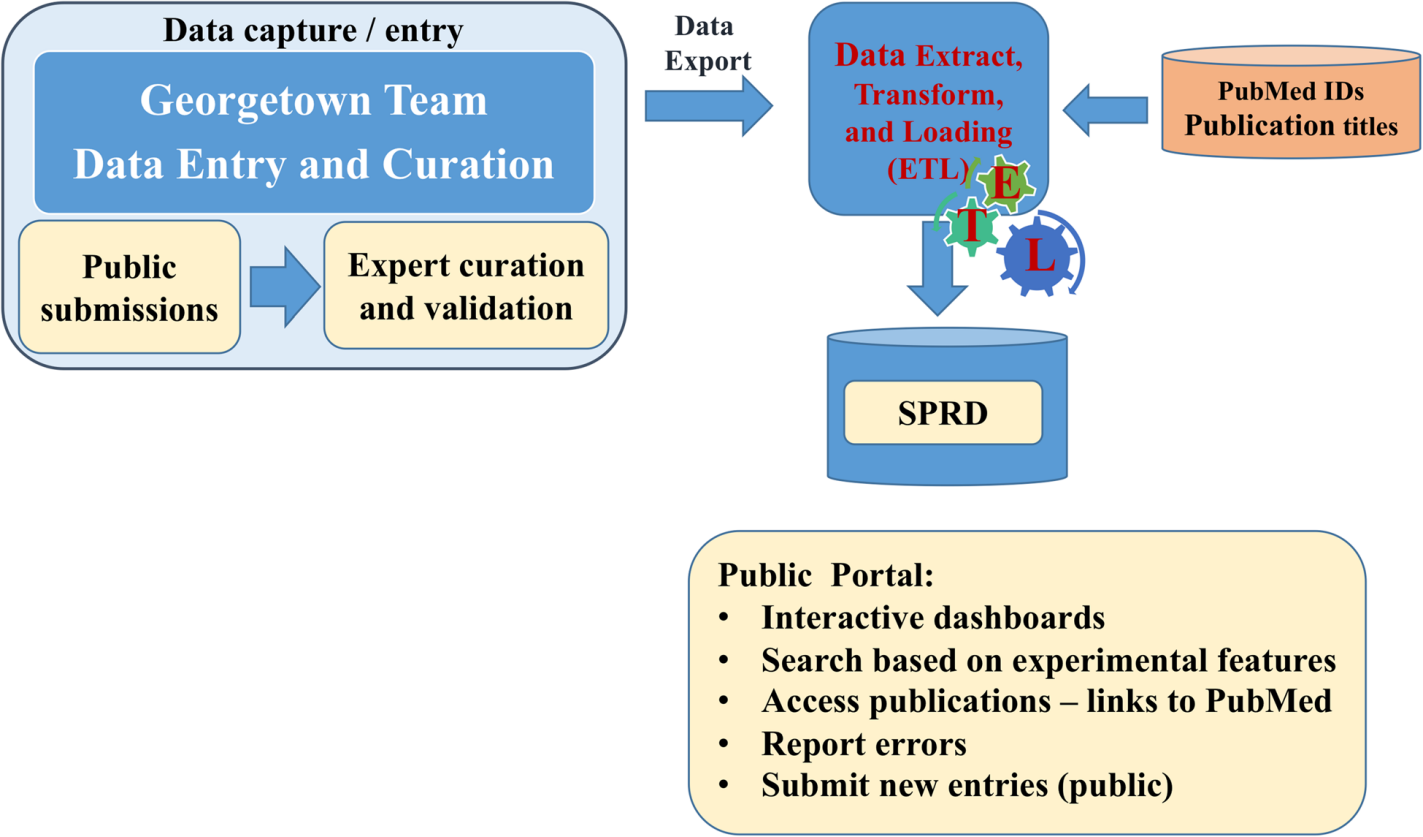
SPRD information workflow

Figure 1 illustrates the workflow and different components developed and implemented for the project. The portal can be accessed via http://www.sprdatabase.info.

#### Data collection

To collect and manage SPRD data entries, we designed and implemented data collection instruments hosted and managed in the Georgetown REDCap based system, a secure, web-based application designed exclusively for building and managing online surveys, study data management and monitoring for research studies. REDCap was developed by Vanderbilt University and the REDCap Consortium, a collection of 4683 institutions from 139 countries currently utilizing the software and contributing to its continuing enhancement and maintenance [35]. REDCap is an easy-to-use, and secure method of flexible yet robust data collection. We manually entered and curated data from 5140 manuscripts found in PubMed and implemented a capability for the public to submit new entries that will be validated by the team before publication in the portal.

We developed Extract, Transform, and Load procedures to copy data from REDCap entries and PubMed sources into a cloud based destination database, which represents the data in a structured searchable format hosted in the Google Cloud Platform (GCP). We leveraged PyMed [36], a Python library that provides access to PubMed through the PubMed API using Python, to retrieve data and links from PubMed.

All SPRD data are hosted and managed in the Georgetown University Cloud-based Virtual Research Environment (VRE), leveraging the Google Cloud Platform (GCP) for provisioning computing resources, securely storing and sharing data. The VRE was designed and developed to overcome barriers met by the research community while complying with institutions’ policies and current state and federal policies and regulations. It is a multi-mission platform that can facilitate the advancement of science, education, and services and will enable the SPRD and investigators to participate in and share data, information and knowledge with the community and research networks. Users can visit www.sprdatabase.info to access the database for the information discussed above. The webpage, especially different tabs within the blue stripe at the left side of the webpage, guides the users to access information included in the database. Clicking “more here” in the search results that is obtained by accessing “Search the database” tab provide access to additional details about the particular entry. At the time of this manuscript submission, we recorded information from 5500 REDCap database entries. We will be updating the database by adding new publications in the future and we encourage users to submit their data as well.

**Fig. 2:**
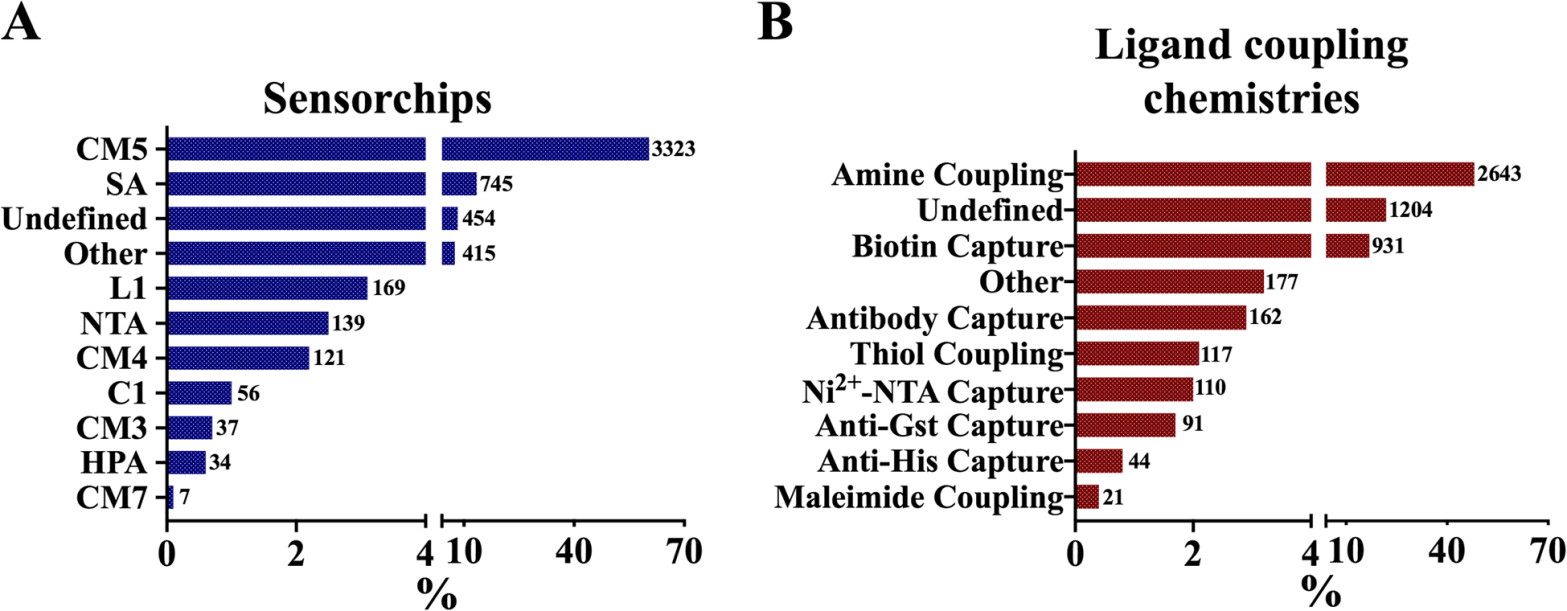
Distribution of most commonly used sensorchips (**a**) and ligand coupling chemistries (**b**) are shown in bar graphs. If the chip type or coupling chemistry was not stated in the original manuscript, it was assigned to “Undefined” group. Numbers next to bars represent the sample size

The 5500 entries we collected from 5140 publications so far had a wide range of experimental conditions, which were most likely optimized for the specific analyte-ligand conditions. CM5 was the mostly used chip (~ 60%) as compared to other types of sensorchips (Fig. 2a). Amine coupling chemistry was the most preferred method (~ 48%) of ligand immobilization (Fig. 2b). This observation indicates that CM5 chip and amine coupling chemistry were the most commonly used sensorchip type and ligand coupling chemistry, respectively. We also observed that “Biacore instruments” were the most frequently used SPR instruments (~ 88%) and the “Biacore 3000” was the most commonly used instrument (~ 40%). Moreover, “protein” was the leading class of ligand (~ 58%) and analyte (~ 56%) as compared to other ligand and analyte classes.

#### Future work

As we launch SPRD with an initial repository of 5500 complete and curated data entries, we anticipate it to grow into a much larger, freely available and trusted knowledge base with up to date information. The envisioned system will require the development of a framework that enables custom design and can scale to accommodate additional workflows, new potential data sources and collaboration options a custom developed web application coupled with a database system can enable new advanced features to design and implement custom developed forms, visualization and reporting capabilities.

Roadmap and future development will consider the automation of data ingestion from different sources and data export to SPRD data repository. We can leverage the available REDCap application programming interface (API), a RESTful web service for storing and retrieving data to and from REDCap. We will survey users, data custodians and the community for feedback and desired new features.

## Conclusions

In summary, the SPRD guides users to search for various analyte-ligand pairs or capture methods in the database. The search results will help users to choose different ligand immobilization/capture strategies, optimum buffer for immobilization, and surface regeneration conditions for a particular ligand. Therefore, the SPRD can be very helpful to efficiently design and execute SPR experiments both for experienced and novice SPR users. We would like to state that our database doesn’t cover entire SPR-based publications so far, however, we will continue to add more information in the future. Our database also offers users an opportunity to correct the mistakes in the SPRD and to enter information from their own or other’s publications that are not included in the database. We would like to request users to notify us if they find errors in the recorded information so that we can correct it in the database. These functionalities can be accessed at the SPRD webpage by clicking “Report a mistake in the database” tab to report mistakes and “Add a new entry to the database” to enter information from their own or other’s publication that are not included in the database.

## Data Availability

The users can access the SPR experimental details we collected for a particular ligand-analyte pair of interest from the SPRD. The users can also have access to the original publication for more detailed information such as reagents, sources, other ligands and analytes. We have provided a reference to each publication that we recorded in the SPRD.
